# Zona atypique chez un patient immunodéprimé

**DOI:** 10.11604/pamj.2013.16.8.3181

**Published:** 2013-09-10

**Authors:** Amal Taghy, Badreddine Hassam

**Affiliations:** 1 Service de Dermatologie-Vénérologie CHU Ibn Sina, Maroc / Faculté de Médecine et de Pharmacie Med V Souissi, Rabat, Maroc

**Keywords:** zona, immunodéprimé, HIV, shingles, immunocompromised, HIV

## Image en médecine

Le zona chez l'immunodéprimé peut prendre un aspect inhabituel, volontiers ulcéro-nécrotique. Il peut également disséminer au niveau cutané ou viscéral ou être d’évolution prolongée ou récidivante. Le risque de surinfection est également accru sur ce terrain. En effet, Le rôle de l'immunité à médiation cellulaire dans l'immunité antivirale est prouvé. Ainsi, les sujets présentant un déficit de l'immunité cellulaire sont susceptibles de développer des formes plus sévères ou atypiques dans leur présentation clinique La prise en charge repose sur l'hospitalisation. Un traitement par Aciclovir par voie IV pendant 7 à 10 j à raison de 10 mg/kg/8 h doit être débuté dans les 48 à 72H. Les antalgiques classiques sont peu efficaces pour le traitement des algies post-zostériennes. La carbamazépine peut être utilisée, en particulier dans les algies trigéminées. La gabapentine aurait un effet antalgique et également sur la restauration du sommeil. Ce sont les antidépresseurs tricycliques qui semblent constituer la meilleure indication à condition qu'ils soient prescrits précocement. On réserve les opiacés par voie orale en cas de douleur persistante. Nous rapportant le cas d'un homme de 45 ans, sous Cyclophosphamide pendant 6 mois pour granulomatose de Wegener, admis pour un placard érythémateux, nécrotique, suintant, hyperalgique, prenant tout le territoire du nerf V1 gauche, parsemé de lésions vésiculo-bulleuses par endroits, un œdème palpébral gauche, des adénopathies sous mandibulaires bilatérales, une fièvre chiffrée à 38° et une altération de l’état général. Le patient a bénéficié d'un traitement par Valex 500mg 2 fois par jour pendant plus de 15 jours et d'antidépresseurs tricycliques avec une évolution lentement favorable. Le zona chez l'immunodéprimé peut prendre un aspect inhabituel, volontiers ulcéro-nécrotique. Il peut également disséminer au niveau cutané ou viscéral ou être d’évolution prolongée ou récidivante. Le risque de surinfection est également accru sur ce terrain. En effet, Le rôle de l'immunité à médiation cellulaire dans l'immunité antivirale est prouvé. Ainsi, les sujets présentant un déficit de l'immunité cellulaire sont susceptibles de développer des formes plus sévères ou atypiques dans leur présentation clinique La prise en charge repose sur l'hospitalisation. Un traitement par Aciclovir par voie IV pendant 7 à 10 j à raison de 10 mg/kg/8 h doit être débuté dans les 48 à 72H. Les antalgiques classiques sont peu efficaces pour le traitement des algies post-zostériennes. La carbamazépine peut être utilisée, en particulier dans les algies trigéminées. La gabapentine aurait un effet antalgique et également sur la restauration du sommeil. Ce sont les antidépresseurs tricycliques qui semblent constituer la meilleure indication à condition qu'ils soient prescrits précocement. On réserve les opiacés par voie orale en cas de douleur persistante. Nous rapportant le cas d'un homme de 45 ans, sous Cyclophosphamide pendant 6 mois pour granulomatose de Wegener, admis pour un placard érythémateux, nécrotique, suintant, hyperalgique, prenant tout le territoire du nerf V1 gauche, parsemé de lésions vésiculo-bulleuses par endroits, un &oelig;dème palpébral gauche, des adénopathies sous mandibulaires bilatérales, une fièvre chiffrée à 38° et une altération de l’état général. Le patient a bénéficié d'un traitement par Valex 500mg 2 fois par jour pendant plus de 15 jours et d'antidépresseurs tricycliques avec une évolution lentement favorable.

**Figure 1:**
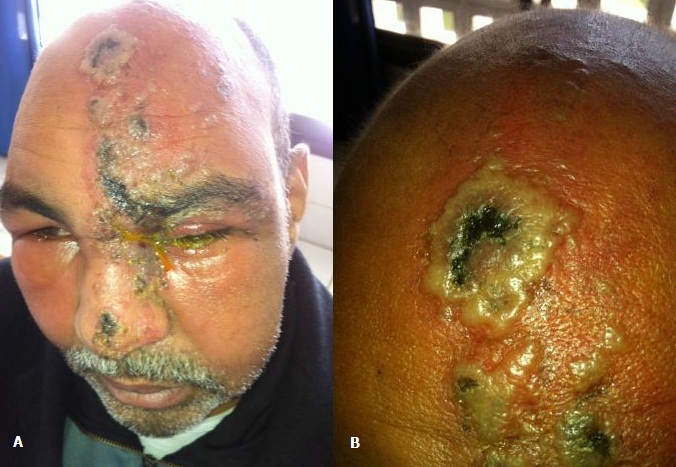
A) Zona nécrotico- bulleux, suintant, prenant le territoire du trijumeaux 1 gauche avec œdème palpébral; B) Lésions nécrotico-bulleuses de l'hémi-front gauche parsemées de croutes mélicériques.

